# Intra-patient comparison of trapeziectomy with LRTI and dual mobility prosthesis for trapeziometacarpal osteoarthritis: a multicenter observational study

**DOI:** 10.1007/s00590-025-04441-y

**Published:** 2025-07-29

**Authors:** Francesco Smeraglia, Enrico Carità, Giulia Frittella, Federico Tamborini, Lorenzo Diaz, Alberto Donadelli, Matteo Guzzini

**Affiliations:** 1 University of Naples Federico II, Naples, Italy; 2 Clinica San Francesco, Verona, Italy; 3 Agostino Gemelli University Polyclinic, Rome, Italy; 4 University of Insubria, Varese, Italy; 5 Università Campus Bio-Medico, Rome, Italy; 6 Saint Camillus International University of Health and Medical Sciences, Rome, Italy

**Keywords:** Trapeziometacarpal osteoarthritis, Arthroplasty, Prosthesis, Dual mobility, Trapeziectomy

## Abstract

**Purpose:**

Thumb osteoarthritis is a debilitating condition that affects a large portion of the elderly population. Conservative treatments for this condition often fail, and a surgical solution is required. Many different surgical techniques have been described, but the current literature has not yet demonstrated the superiority of one over the others. In this study, we analyzed the clinical and radiological findings of a population of 26 patients who were operated on both hands but with different techniques.

**Methods:**

One hand underwent trapeziectomy with suspension arthroplasty, while the other hand was operated on with a double mobility trapeziometacarpal prosthesis.

**Results:**

Our findings show that, while on the long-term follow-up the two techniques are equally valid, in the short term, the hands that were operated on with the prosthesis had a faster recovery of strength and pain.

**Conclusion:**

Therefore, we reckon that double mobility trapeziometacarpal prostheses are a better choice of treatment, especially for patients who require a faster recovery for work or leisure activities.

## Introduction

Osteoarthritis (OA) of the trapeziometacarpal (TM) joint is a common and debilitating condition, especially among postmenopausal women, that significantly impairs hand function and negatively affects quality of life. Conservative management, including splinting, physical therapy, and corticosteroid injections may offer temporary relief, but surgical intervention is often required in cases of persistent pain and functional limitations [1].

When conservative treatment fails, several surgical techniques have been proposed, but none has been proven superior to another [2]. LRTI has long been considered the gold standard due to its reproducibility and long-term effectiveness, though it is often associated with prolonged recovery, gradual thumb shortening and the consequent loss of strength may represent an important drawback of the technique, particularly in young and highly demanding populations [2, 3].

Conversely, total joint arthroplasty represents a promising alternative, as it helps maintain the length of the first ray, thereby enhancing pinch strength and allowing full range of motion. Although various prosthetic designs have been introduced and discussed in the literature, no definitive gold standard has been established to date [2, 4, 5].

Dual mobility prostheses, on the other hand, aim to restore thumb biomechanics more anatomically, with faster return to function and preservation of bone stock, but concerns persist regarding implant survivorship and technical complexity [6, 7].

To date, few studies have directly compared the outcomes of LRTI and dual mobility prosthesis in a controlled setting. The present study offers a unique intra-patient comparison, as each patient received one technique for hand, thereby minimizing confounding variables such as age, sex, activity level, and systemic comorbidities.

The aim of this multicentre, retrospective study is to compare the clinical and radiographic outcomes of LRTI, described by Altissimi [8], versus dual mobility prosthesis for TM osteoarthritis within the same patients. We hypothesize that TM prosthesis will lead to faster pain relief and functional recovery compared to LRTI.

## Materials and methods

### Study design and participants

This multicentre, retrospective observational study compared the outcomes in both hands of patients who had previously undergone trapeziectomy with LRTI on one hand for trapeziometacarpal osteoarthritis (TM OA), and later developed the same condition in the contralateral hand, for which they underwent total joint replacement using a dual mobility prosthesis. All surgical procedures—including the initial trapeziectomy with LRTI, performed between 2017 and 2022, and the subsequent total joint replacement, performed between January 2020 and July 2022—were carried out by the same group of Level 4 hand surgeons [9].

Inclusion criteria were severe pain at the base of the thumb and limitation of normal daily activities, which did not respond to at least three months of conservative therapy, such as splinting, systemic anti-inflammatories, local corticosteroid injections or physical therapy, and thenar eminence strengthening exercises. Regarding radiographic evidence, the inclusion criteria were OA of the TM joint with stages II and III according to Eaton-Littler [10] classification. The exclusion criteria encompassed the presence of scaphotrapeziotrapezoid (STT) OA (IV according to Eaton-Littler), poor trapezium bone quality as depicted on the pre-operative radiograph, and ongoing treatment with systemic corticosteroids for underlying diseases. The aforementioned exclusion criteria were only applicable to the joint replacement group.

The study was in line with the Ethical Standards of the 1975 Declaration of Helsinki, as revised in 2008.

All patients were extensively informed about the surgical procedure, and valid consent was collected for the surgery and participation in the study.

### Operative techniques and prosthesis

The procedures were performed under brachial plexus block and a tourniquet was applied. A first-generation cephalosporin was given intravenously as per pre-operative prophylaxis guidelines.

Trapeziectomy with LRTI was performed as described by Altissimi [8]: after performing the trapeziectomy, a four cm long string of the Flexor Carpi Radialis (FCR) was harvested and secured to the base of the I metacarpal bone with a miniature bone anchor.

Regarding the hands that were operated on with double mobility TM prosthesis, two types of prostheses were implanted, both press-fit, modular, and dual mobility: Touch® prosthesis (Kerimedical; route des Acacias, Les Acacias, Switzerland) and Maïa™ prosthesis (Lépine Group; rue Jacquard, Genay, France).

The procedure was performed using a dorsoradial approach. A longitudinal skin incision was made along the course of the extensor pollicis brevis (EPB) tendon. The capsule was opened longitudinally. An approximately 5 mm perpendicular osteotomy was made at the base of M1 using an oscillating saw. The first metacarpal base was sub-dislocated to allow trial prosthesis insertion.

Marginal osteophytes were removed to improve joint congruity. Correct guidewire positioning was assessed by radiographs in 2 planes. Dedicated reamers were used to prepare the trapezium. The prosthetic cup was implanted perpendicular to the longitudinal axis of the first metacarpal.

The optimal neck length was determined by evaluating tendon tension using the tenodesis effect. If excessive tightness was observed, the neck length was adjusted to avoid over-tensioning. Closure was performed suturing the capsule, followed by layered skin closure using absorbable sutures.

### Post-operative care

The hands operated with trapeziectomy and LRTI were immobilized for three post-operative weeks with a long thumb spica cast or brace. Wound dressing was performed after one week and stitch removal was performed after two weeks. After removing the cast, an average of three months of rehabilitation was prescribed to regain the full range of motion (ROM) of the thumb and hand grip strength of the hand.

As for the hands that underwent joint replacement, during the first week, they were protected by wrist bandaging with the inclusion of the first finger, which allowed but limited the mobility of the TM and the metacarpophalangeal joints. After one week, wound dressing and application of a patch were performed, which allowed the patient to achieve full ROM of the TM. The stitches were removed after two weeks, and the patient was allowed to return gradually to his daily activities.

### Clinical and radiographic evaluations

All patients, for both procedures, were assessed pre-operatively and post-operatively at 1, 3, 6, 12, and 24 months.

The clinical follow-up was the same for all patients. It consisted of an assessment of pain and functional capacity in daily activities. Visual Analogical Scale (VAS) and Disability of the Arm, Shoulder, and Hand (DASH) scores were submitted to all patients. To assess hand mobility, specifically ROM, the Kapandji Test was performed as well as the ROM in radial abduction (measured with a goniometer while the ulnar side of the patient’s forearm and hand were positioned on the table).

Moreover, hand strength was measured through the utilization of the Jamar dynamometer, performing tests such as Hand Grip, Tip Pinch, and Key Pinch. We evaluated the patient’s satisfaction with a five-step Likert-type scale (excellent—very good—good—fair—poor).

The radiological follow-up consisted of radiographs in the two standard projections of both hands: lateral and anteroposterior. As for the hands operated with trapeziectomy and LRTI, they were evaluated by measuring the thumb length to detect and quantify any subsidence of the I metacarpal on the scaphoid. Regarding the prostheses, the scoring protocol described by Lussiez [11] et al. was used to assess the general radiographic state of the implants. This score analyses the presence of subchondral cysts, tilted cup, and implant dislocation, it classifies both cup and stem migration and loosening into four progressive stages.

### Statistical analysis

Data collected during follow-up from hands treated with the two different techniques were compared with pre-operative data and then between the two techniques for each observation interval. Statistical analyses were conducted using analysis of variance (ANOVA) for repeated measures to assess the evolution of VAS and DASH scores over time within each surgical group (prosthesis and suspension arthroplasty). Pairwise comparisons between follow-up timepoints (preoperative, 3 months, 6 months, 12 months, and 24 months) were performed using Bonferroni correction to adjust for multiple testing. A p-value < 0.05 was considered statistically significant. Homogeneity of variances was tested using Bartlett’s test. All statistical analyses were performed by an independent statistician using Stata software.

## Results

### Patients

This multicentre study involved 26 patients, who fulfilled the inclusion and exclusion criteria. The total number of hands operated on was 52, of which 26 underwent the trapeziectomy with LRTI and 26 the TM total joint replacement with a dual-cup mobility prosthesis. Of the 26 hands treated with trapeziectomy and LRTI, 14 were dominant and 12 were non-dominant. Among the 26 hands that underwent total joint replacement, 12 were dominant and 14 were non-dominant.

All data about patients are summarized in Table 1.

**Table 1:** Characteristics of the patient cohort

Parameters	Total patients, 26
Age, mean ± (range), years	61.23 ± 8.2 (45–79)
Gender, n (%)	
Females	23 (88.46)
Males	3 (11.54)
Eaton-littler stage	
Prosthesis	9 (Stage II) 17 (Stage III)
Trapeziectomy with LRTI	6(Stage II) 20 (Stage III)
Dominance, n (%)	
Prosthesis	12 (46.15)
Trapeziectomy with LRTI	14 (53.85)
Prosthesis implanted, n (%)	
Touch	12 (46.15)
Maïa	14 (53.85)

SD = standard deviation; n = number

### Pain and hand function

**Fig. 1:**
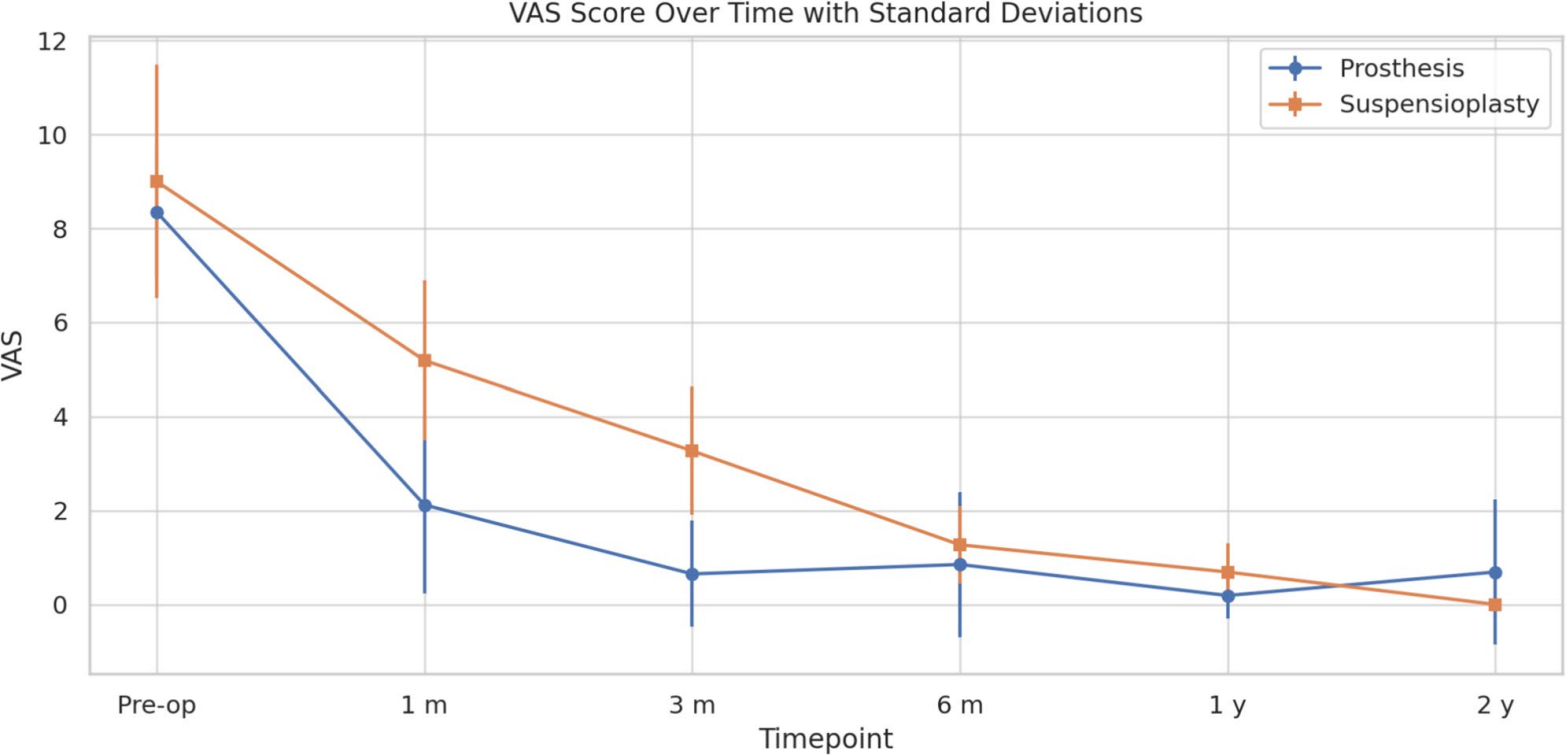
VAS score over time with standard deviation

ANOVA revealed a significant difference between pre-operative and 2-year follow-up values for VAS (p < 0.0001). The Bonferroni correction demonstrated that in the prosthesis groups the greatest improvement in pain (VAS) occurred between pre-op and 3 m (p < 0.001) and between 3 and 6 m (p = 0.004). In the suspensioplasty group such improvement also continued between 6 and 12 m (p < 0.001). ANOVA confirmed a statistically significant reduction over time in both groups (p < 0.0001), with earlier improvement observed in the prosthesis group. (Fig. 1).

**Fig. 2:**
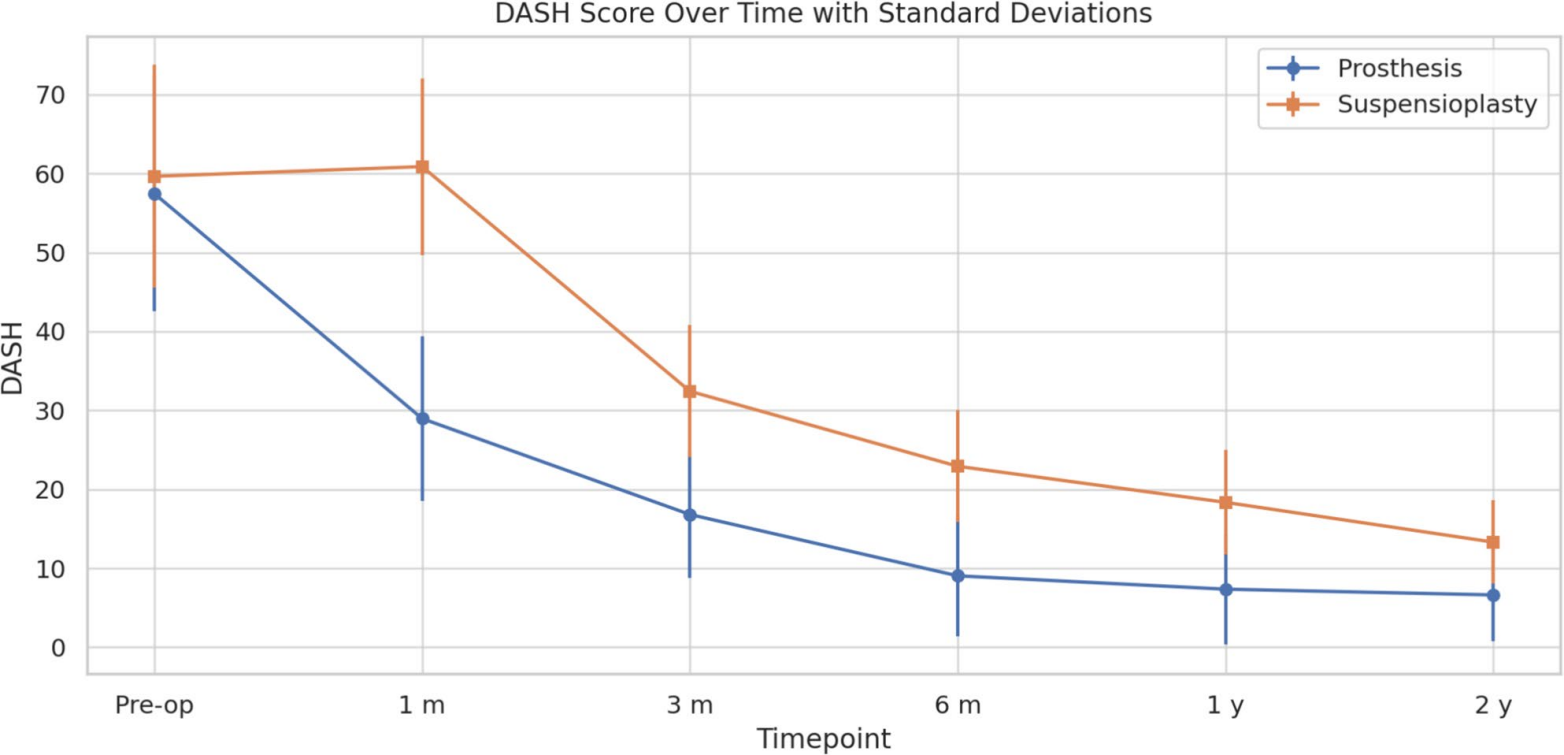
DASH score over time with standard deviation

ANOVA revealed a significant difference between pre-operative and 2-year follow-up values for DASH (p < 0.0001). The Bonferroni correction demonstrated that in the prosthesis groups the greatest improvement in function (DASH) occurred between pre-op and 3 m (p < 0.001) and between 3 and 6 m (p < 0.001. In the suspensioplasty group the improvement in function was observed later than 3 m, then continuing up to 12 m (p < 0.001). These findings suggest that although both techniques yield excellent long-term functional outcomes, total joint replacement with a dual mobility prosthesis offers a significantly faster recovery in the early postoperative period (Fig. 2).

### Handgrip, key pinch and pulp pinch strength

Progressive improvement was observed over time for all grip and pinch strength parameters in both treatment groups. Handgrip strength showed significant main effects of time (p < 0.001, η^2^ = 0.789) and treatment (p < 0.001, η^2^ = 0.367), with patients in the prosthesis group consistently outperforming those in the suspension group. The difference was most pronounced at 3 and 6 months, with the gap narrowing at 24 months (27.3 kg vs. 24.4 kg). The interaction between time and treatment was statistically significant but modest (p = 0.001, η^2^ = 0.077), indicating a slightly steeper recovery trajectory in the prosthesis group.

For key pinch strength, the analysis revealed a statistically significant effect of time (p = 0.025, η^2^ = 0.510); however, neither the main effect of treatment (p = 0.190) nor the interaction with time (p = 0.298) reached statistical significance, likely due to the small sample size. Nevertheless, patients treated with a prosthesis generally exhibited higher key pinch values throughout the follow-up period, particularly at the initial stages.

Similarly, pulp pinch strength improved significantly over time (p = 0.037, η^2^ = 0.490), with a significant main effect of treatment (p = 0.041, η^2^ = 0.689). Prosthesis patients demonstrated superior performance, particularly at 1 and 3 months. However, the interaction between time and treatment was not significant (p = 0.353), suggesting similar recovery trends across both groups. Notably, the pulp pinch score at one month was significantly higher in the prosthesis group (4.0 kg versus 1.75 kg).

These findings emphasise the faster recovery of pinch and grip function following total joint replacement, despite similar long-term outcomes for both surgical strategies.

### Kapandji

Kapandji scores improved significantly over time in both groups (p < 0.001), with a faster recovery observed in the prosthesis group. At 1 and 3 months, patients treated with a prosthesis achieved higher opposition scores (mean 9.19 and 9.58) compared to the suspension group. However, by 12 and 24 months, both groups reached similarly high scores (9.69 vs 9.96, p = n.s.), indicating excellent long-term thumb opposition regardless of technique. The interaction between time and treatment was significant (p < 0.001), confirming a steeper early improvement in the prosthesis group.

### Satisfaction

Regarding satisfaction with the TM prosthesis, 19 patients (73.1%) rated the outcome as excellent and 7 (26.9%) as very good. In contrast, satisfaction following trapeziectomy with LRTI was lower: 4 patients (15.4%) rated the result as excellent, 10 (38.5%) as very good, and 12 (46.1%) as good at the latest follow-up.

Table 2 provides a comprehensive summary of all mean values and standard deviations for each outcome measure at the different timepoints.

**Table 2:** Outcome values

Outcome	Group	Pre-op	1 m	3 m	6 m	1 y	2 y
VAS	Prosthesis	8.35 (1.41)	2.12 (1.88)	0.65 (1.13)	0.85 (1.54)	0.19 (0.49)	0.69 (1.54)
	Suspensionplasty	9.00 (2.48)	5.19 (1.70)	3.27 (1.37)	1.27 (0.83)	0.69 (0.62)	0.00 (0.00)
DASH	Prosthesis	57.46 (14.89)	28.96 (10.47)	16.81 (8.09)	9.04 (7.62)	7.35 (6.98)	6.62 (5.87)
	Suspensionplasty	59.65 (14.10)	60.88 (11.18)	32.42 (8.38)	22.92 (7.08)	18.35 (6.64)	13.31 (5.27)
Handgrip (kg)	Prosthesis	14.42 (2.89)	12.88 (3.10)	17.27 (3.95)	20.00 (3.58)	26.73 (5.05)	27.31 (5.10)
	Suspensionplasty	13.50 (3.00)	9.88 (1.80)	11.31 (2.04)	15.08 (2.97)	22.35 (4.80)	24.42 (5.13)
Key Pinch (kg)	Prosthesis	3.75 (0.96)	4.00 (1.16)	4.00 (1.16)	4.00 (1.16)	4.25 (0.96)	4.75 (0.96)
	Suspensionplasty	3.00 (0.00)	2.00 (0.00)	2.50 (0.71)	3.00 (0.00)	3.50 (0.71)	5.00 (1.41)
Pulp Pinch (kg)	Prosthesis	3.00 (1.41)	4.00 (1.41)	5.00 (1.41)	4.00 (0.00)	4.50 (0.71)	5.00 (0.00)
	Suspensionplasty	2.75 (0.96)	1.75 (0.50)	3.25 (0.50)	3.25 (0.50)	3.75 (0.96)	3.75 (0.96)
Kapandji	Prosthesis	8.50 (1.00)	9.19 (0.75)	9.58 (0.58)	9.50 (0.64)	9.73 (0.52)	9.69 (0.58)
	Suspensionplasty	8.54 (0.88)	8.65 (0.85)	9.00 (0.82)	9.31 (0.78)	9.92 (0.28)	9.96 (0.19)

### Radiological findings

All prostheses showed good osteointegration and there were no complications in terms of dislocation of the cup and/or stem, migration of the same components, or lucent zones of the cup or metacarpal bone. None of the hands operated on with trapeziectomy and LRTI showed a significant decalage of the first ray.

### Complications

No major or minor complications were observed.

## Discussion

This study aimed to compare the mid-term results of two different surgical techniques performed on the same patients, operating one hand with trapeziectomy and LRTI and the contralateral hand with the TM joint replacement with a dual mobility prosthesis.

At 24 months of follow-up, our study shows good outcomes of the trapeziectomy LRTI technique. In a recent study by Altissimi [8] on 299 patients, similar findings to ours were observed: VAS at follow-up was 1–3 in 84% of patients, Quick DASH improved from 52 pre-operatively to 20 post-operatively and the Kapandji scale was 9–10 in 76% of hands.

The systematic review by Villari [12] provides comprehensive insight into the outcomes and complication rates of dual mobility implants. The review, which encompassed 931 patients across ten studies, documented a failure rate of 2.7%, with dislocation and aseptic loosening identified as the predominant indications for revision surgery. In a similar vein, the present series documented a survival rate of 100% at 2-years follow up.

Falkner et al. [13] conducted a prospective comparative study contrasting dual mobility prostheses against resection arthroplasty. The findings of the study highlighted that patients who received dual mobility implants experienced a more rapid postoperative recovery, exhibited superior pinch strength, and demonstrated significantly improved QuickDASH and Michigan Hand Questionnaire (MHQ) scores. These results are closely aligned with our own data.

With regard to the range of motion, the enhancements observed in the Kapandji score are consistent with those documented in the systematic review [12] and Frey et al. [14], thereby substantiating the efficacy of the prosthesis in restoring thumb mobility. Furthermore, the enhancement in pinch and grip strength observed in the patient cohort lends further credence to the findings of the systematic review's meta-analysis, underscoring the functional gains facilitated by dual mobility arthroplasty.

Excessive lengthening of the thumb column has been associated with an increased risk of developing De Quervain's tenosynovitis [15]. However, in the present series, no cases of tendinitis were observed. This finding indicates that ensuring appropriate restoration of thumb length, by means of adequate tensioning of the thenar musculature, is of crucial importance. This is because it not only enhances thumb abduction, but also contributes to the correction of MCP joint hyperextension and Z-deformity, which frequently eliminates the need for additional procedures at the MCP level.

The extant evidence suggests that, within the initial five-year period, dual mobility prostheses exhibit a reduced incidence of dislocations and complications in comparison to single mobility designs [16]. However, the long-term sustainability of these favourable results remains uncertain, and conclusive evidence demonstrating the superiority of dual mobility implants over single mobility systems is still lacking [12].

This study has several strengths. Firstly, the intra-patient design eliminates inter-subject variability, enabling more accurate comparisons between the two surgical techniques by controlling for confounding factors such as age, sex, handedness, occupation, pain tolerance and systemic comorbidities. To our knowledge, this is one of the few studies to directly compare LRTI and dual mobility prostheses within the same patient cohort, thereby enhancing internal validity. Furthermore, all procedures were performed by experienced Level 4 hand surgeons in specialised centres, ensuring consistency in surgical technique and postoperative care.

However, some limitations must be acknowledged. The retrospective nature of the study carries an inherent risk of selection and reporting bias. Although the sample size is adequate for an observational comparison, it remains limited, which may affect the statistical power of subgroup analyses. Furthermore, while intra-patient comparisons control for many variables, they introduce potential biases related to the sequence of surgeries and the possible influence of the first surgical outcome on the patient’s perception of the second. Finally, the relatively short follow-up period prevents definitive conclusions from being drawn about the long-term survivorship of the prosthetic implants. However, we plan to continue monitoring this patient cohort with annual follow-up visits in order to assess long-term outcomes and implant survival over time.

The findings of this study suggest that the TM prosthesis is a beneficial surgical option for the treatment of OA TM. The TM joint replacement has been shown to facilitate a more expeditious recovery of pain and hand function following surgery when compared with trapeziectomy with LRTI. This demonstrates a substantial enhancement in the patient's quality of life and a more expeditious return to their daily activities. Consequently, the TM prosthesis is recommended as the primary surgical intervention for patients diagnosed with stage II or III Eaton-Littler OA. In regard to recovery procedures following prosthesis or stage IV OA, the prevailing opinion is that trapeziectomy remains the optimal choice.

### AI use statement

Language editing and reference formatting were assisted by a large language model (ChatGPT, OpenAI). All scientific content and conclusions have been critically reviewed and validated by the authors, who take full responsibility for the manuscript’s integrity and accuracy.

## Data Availability

No datasets were generated or analysed during the current study.
